# Effects of *in situ* Remediation With Nanoscale Zero Valence Iron on the Physicochemical Conditions and Bacterial Communities of Groundwater Contaminated With Arsenic

**DOI:** 10.3389/fmicb.2021.643589

**Published:** 2021-03-17

**Authors:** Ana Castaño, Alexander Prosenkov, Diego Baragaño, Nerea Otaegui, Herminio Sastre, Eduardo Rodríguez-Valdés, José Luis R. Gallego, Ana Isabel Peláez

**Affiliations:** ^1^Area of Microbiology, Department of Functional Biology and Environmental Biogeochemistry and Raw Materials Group, University of Oviedo, Oviedo, Spain; ^2^INDUROT and Environmental Biogeochemistry and Raw Materials Group, Campus of Mieres, University of Oviedo, Mieres, Spain; ^3^TECNALIA, Basque Research and Technology Alliance (BRTA), Parque Tecnológico de Bizkaia, Derio, Spain; ^4^Department of Chemical and Environmental Engineering and Environmental Biogeochemistry and Raw Materials Group, University of Oviedo, Oviedo, Spain; ^5^University Institute of Biotechnology of Asturias (IUBA), University of Oviedo, Oviedo, Spain

**Keywords:** zero-valent iron, nanoparticles, groundwater, arsenic, *in situ* remediation, ecotoxicity, bacteria

## Abstract

Nanoscale Zero-Valent Iron (nZVI) is a cost-effective nanomaterial that is widely used to remove a broad range of metal(loid)s and organic contaminants from soil and groundwater. In some cases, this material alters the taxonomic and functional composition of the bacterial communities present in these matrices; however, there is no conclusive data that can be generalized to all scenarios. Here we studied the effect of nZVI application *in situ* on groundwater from the site of an abandoned fertilizer factory in Asturias, Spain, mainly polluted with arsenic (As). The geochemical characteristics of the water correspond to a microaerophilic and oligotrophic environment. Physico-chemical and microbiological (cultured and total bacterial diversity) parameters were monitored before and after nZVI application over six months. nZVI treatment led to a marked increase in Fe(II) concentration and a notable fall in the oxidation-reduction potential during the first month of treatment. A substantial decrease in the concentration of As during the first days of treatment was observed, although strong fluctuations were subsequently detected in most of the wells throughout the six-month experiment. The possible toxic effects of nZVI on groundwater bacteria could not be clearly determined from direct observation of those bacteria after staining with viability dyes. The number of cultured bacteria increased during the first two weeks of the treatment, although this was followed by a continuous decrease for the following two weeks, reaching levels moderately below the initial number at the end of sampling, and by changes in their taxonomic composition. Most bacteria were tolerant to high As(V) concentrations and showed the presence of diverse As resistance genes. A more complete study of the structure and diversity of the bacterial community in the groundwater using automated ribosomal intergenic spacer analysis (ARISA) and sequencing of the 16S rRNA amplicons by Illumina confirmed significant alterations in its composition, with a reduction in richness and diversity (the latter evidenced by Illumina data) after treatment with nZVI. The anaerobic conditions stimulated by treatment favored the development of sulfate-reducing bacteria, thereby opening up the possibility to achieve more efficient removal of As.

## Introduction

Nanoscale zero-valent iron (nZVI) is the most widely used nanomaterial for soil and groundwater remediation in Europe and the United States (Mueller et al., 2012; Bardos et al., 2015). This material is effective on a wide range of contaminants, including halogenated compounds, nitrate, phosphate, polycyclic aromatic hydrocarbons and metal(loid)s (Jiang et al., 2018; Pasinszki and Krebsz, 2020). Among other characteristics (such as low cost, ease of production and a presumably low impact on the environment, since iron is abundant in the earth’s crust; Jiang et al., 2018), nZVI show high reactivity toward a broad range of pollutants and can be used in the remediation of specific targets (such as aqueous slurries). These properties are attributed to the small size of the nanoparticles, which confers them a high specific surface area and mobility, and to their strong reducing capacity (Cagigal et al., 2018; Pasinszki and Krebsz, 2020). nZVI has some drawbacks, mainly related to its tendency to aggregate, which hinders its supply and mobility in the soil (Stefaniuk et al., 2016). However, aqueous suspensions of this material can be injected directly into groundwater without further excavation, thereby making this treatment highly versatile (Mueller et al., 2012; Jiang et al., 2018).

Nanoscale zero-valent iron treatment of contaminated soils also has other effects that must be considered when evaluating its environmental impact on living beings (humans and other animals, plants and microorganisms); these effects mostly refer to soil health (Stefaniuk et al., 2016). Soil microorganisms (the soil microbiome) form a highly complex community that performs critical activities, including soil-atmosphere gas exchange, processes related to plant growth promotion, responses to adverse environmental conditions, synthesis of bioactive compounds such as antimicrobials, immunity to pathogenic species, and driving biogeochemical cycles through the decomposition and biotransformation of organic matter and pollutants (Zhu et al., 2019; Tian et al., 2020). Microbial biogeochemical activities also include redox transformation of arsenic via As(V) reduction and As(III) oxidation, which directly affect the mobility and bioavailability of this metalloid (Yamamura and Amachi, 2014), and removal of As(V) by nZVI includes adsorption and co-precipitation reactions with Fe(II), or adsorption and oxidation of As(III) to As(V) (Pasinszki and Krebsz, 2020). Finally, microorganisms and nZVI can interact, potentially participating in the oxidation-linked “aging” of nZVI (Xie et al., 2017). Given the growing interest in the effects of nZVI on the microbial populations of the sites subjected to remediation treatment with this material, it is pertinent to carry out toxicity studies in true field conditions, or in a setting that highly resembles these conditions.

Laboratory-scale experiments in activated sludge (Wu et al., 2013), nitrifying sequencing batch reactors (Ma et al., 2015), and other laboratory batch or microcosm approaches (Kirschling et al., 2010; Cullen et al., 2011; Tilston et al., 2013; Saccà et al., 2014; Rónavári et al., 2016; Ševcù et al., 2017; Xue et al., 2018; Gómez-Sagasti et al., 2019; Crampon et al., 2019; Fajardo et al., 2020) allowed analysis of the interaction of soil or water microbial populations and the environmental matrix with nZVI. However, the results from these studies have not provided a clear picture of the toxicity of nZVI as they have shown different effects on the distinct bacterial groups present in the environment. The diversity of the effects is not unexpected as the ecotoxicity of nZVI is determined by the composition of the microbial communities present (which will differ depending on the environment analyzed), the dose of nanoparticles applied, and the parameters potentially affecting the extent of Fe transformation and reactivity, and therefore its mobility and availability (Cagigal et al., 2018; Jiang et al., 2018). This uncertainty extends to the prediction of the effects of *in situ* remediation using nZVI relative to those observed in lab experiments since there is only limited information. However, available *in situ* remediation results point to an attenuated or non-existent toxic effect of nZVI treatments (Němeček et al., 2014; Kocur et al., 2016; Czinnerová et al., 2020).

Here we sought to provide further information on the possible toxic effects of nZVI nanoparticles in the field. To this end, we performed an *in situ* nZVI treatment of groundwater from a brownfield strongly affected by arsenic and other metal (loid) elements and then monitored the changes in the bacterial communities and physico-chemical environmental conditions.

## Materials and Methods

### Site Description

The study site, known as Nitrastur, was one of the main fertilizer plants in Spain until its closure in 1997. The presence of pollutants, such as arsenic, copper, lead and zinc, in soils and groundwater was caused by the use of different waste products as landfill over the quaternary alluvial plain (Gallego et al., 2016). The main waste, pyrite ash, was produced in large amounts by the roasting of sulfide minerals in the sulfuric acid production line of the fertilizer plant. Fluctuations in the water table level allow groundwater to come into contact with the pyrite ash, resulting in the leaching of pollutants into the aquatic environment (Baragaño et al., 2020). The site was subjected to an extensive previous environmental forensic study, which included the analysis of multiple elements of composite samples for risk assessment purposes and the selection of remediation strategies adapted to future land use (Gallego et al., 2016). In the context of an international cooperative project of the European Commission’s FP7 (“Taking Nanotechnological Remediation Processes from Lab Scale to End User Applications for the Restoration of a Clean Environment”, NANOREM)^1^, the site has since been used to develop *in situ* groundwater remediation technologies involving the injection of nanoparticles into the subsoil.

### Application of Zero-Valent Iron Nanoparticles

**FIGURE 1:**
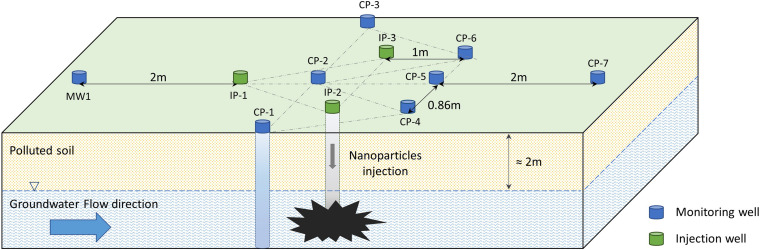
Scheme of the wells excavated in the Nitrastur site for nZVI groundwater treatment. IP, injection wells; CP, monitoring wells.

The commercially available NANOFER STAR (Nanoiron Ltd., Czechia) is a powder of iron oxide-coated stabilized nZVI, with an average particle size of < 50 nm and comprising 65–80% iron (Fe^0^) and 20–35% iron oxide. Eleven wells with a diameter of 7.6 cm were drilled perpendicularly to the groundwater flow on the Nitrastur site (Figure 1). Three of them (IP-1, IP-2 and IP-3) were used to inject nZVI into the groundwater and eight (CP-1, CP-2, CP-3, CP-4, CP-5, CP-6, CP-7, and MW-1) served for monitoring physico-chemical and microbiological parameters before and after treatment. A total of 250 kg of NANOFER STAR nanoparticles in a 10% nZVI suspension was introduced into the injection wells (IPs), and bacterial communities and physico-chemical environmental conditions were subsequently monitored for 6 months.

### Sampling and Monitoring Physico-Chemical Parameters

Water samples for the chemical and microbiological analysis were collected in sterilized plastic vials in triplicate from the eight monitoring wells (CP-1, CP-2, CP-3, CP-4, CP-5, CP-6, CP-7, and MW-1) before the addition of nZVI (time 0), and at 3, 5, 9, 12, 16, 19, 25, 34, 61, 90, 122, 151, and 184 days of the treatment. Before collecting the samples, wells were purged by pumping. Different pump tubes were used for each well to avoid cross-contamination. Samples were collected using a peristatic pump, and parameters such as temperature (T^a^), pH, dissolved oxygen (DO), oxidation-reduction potential (ORP) and electrical conductivity (EC) were measured using a portable multi-analyzer (YSI Professional Plus) coupled to the pump. Samples for metal(loid) concentrations (As, Cd, Cu, Ni, Pb, and Zn) were passed through a 0.45-μm filter and preserved by acidification using HNO_3_. Aliquot subsamples were then subjected to analysis by Inductively Coupled Plasma-Mass Spectrometry (Agilent Technologies 7700 ICP-MS) using Isotope Dilution Analysis. Detection limit for all elements is 0.1 μgL^–1^, except for Cu, Ni, and Zn, which is 0.25 μgL^–1^ (Baragaño et al., 2020). Total dissolved iron and Fe(II) were determined by photometric analysis (NEN 6482). Sulfate concentrations were measured by continuous flow analysis (CFA) and nitrate by spectrophotometric analysis (ISO 22743:2006 and ISO 15923-1: 2013 respectively). The values shown for the individual wells correspond to the average of three measurements with a standard error < 5%.

### Microscopy Analysis of Bacterial Viability

Bacterial viability was analyzed in groundwater samples from CP-5 and CP-6 wells three days after the addition of nZVI. One liter of water was centrifuged at 8,500 *g* and the sediment was resuspended in 1 mL of the same water. Cells were stained with the LIVE/DEAD Bac-Light bacterial viability kit (L-13152; Invitrogen) to detect viable cells (Menendez-Vega et al., 2007). Slides were examined under a Leica TCS SP8X Spectral Confocal Laser Microscope at excitation/emission wavelengths of 490/635nm for propidium iodide (PI) and 480/500 nm for SYTO 9. Images were processed with the Leica Confocal Software. In the presence of double staining, bacterial cells with intact membranes appear green whereas damaged or dead cells appear red. Images of the same samples were also obtained in differential interference contrast mode on the same equipment.

### Isolation and Identification of Cultured Bacteria

**FIGURE 2:**
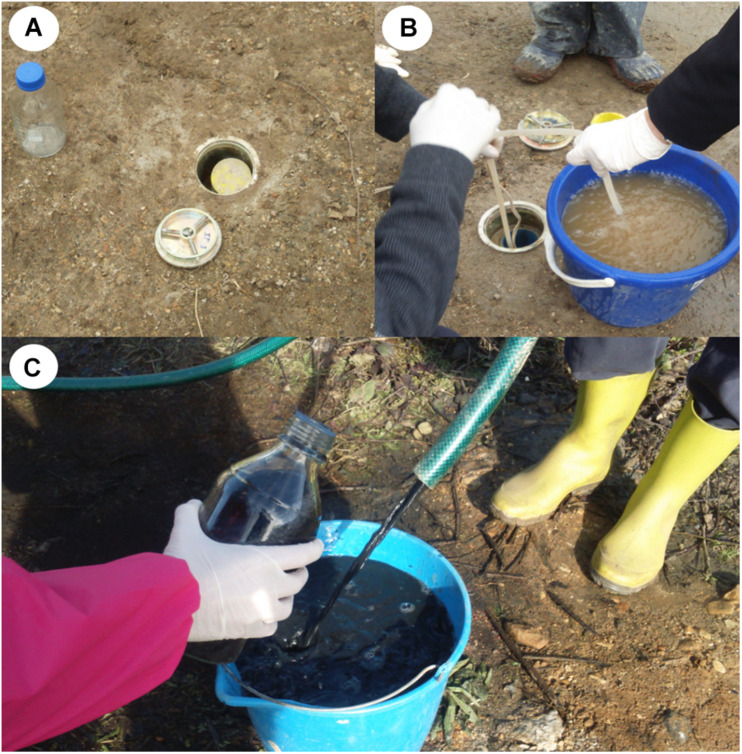
**(A)** Monitoring well excavated in the land. **(B)** Groundwater purged from a well. **(C)** groundwater with nZVI (appearing as a black suspension) collected after purge.

Samples for microbiological analysis were taken from four wells (CP-2, CP-4, CP-5, and CP-6), at different times during the treatment (0, 3, 19, 90, and 184 days after nanoparticle addition). To this end, 1 L of groundwater was collected in sterile bottles after purging and discarding the first 2 L of water (Figure 2). The samples were immediately centrifuged at 8,500 *g* for 15 min, and the sediments were resuspended in 50 mL water. Next, 0.1 ml from 10-fold serial dilutions obtained from each sample were spread on a surface of diluted Tryptic Soy Agar (TSA) medium (Tryptic Soy Broth 1:10 Merck, plus 1% agar) in a Petri dish and incubated at 30°C. Viable bacterial concentration was expressed as a number of colony-forming units per ml of groundwater. Phenotypically different colonies were reseeded in the same medium to obtain pure cultures. To maintain the strains and preserve metabolic capacity, bacterial cells were frozen at −70°C in preserving medium (1.7% wt/vol trehalose and 25% vol/vol glycerol) or lyophilized (1.7% wt/vol trehalose, 2% wt/vol skimmed milk, and 1.25% vol/vol MOPS medium).

For identification, bacterial genomic DNA of the isolates was extracted from the cultured bacteria using the GeneMATRIX Soil DNA Purification Kit (EURx), and bacterial 16S rRNA genes were amplified by PCR in a Minicycle (BioRad) using the primers 27F (5′AGAGTTTGATCCTGGCTCAG 3′) and 1492R (5′ GGTTACCTTGTTACGACTT 3′) with OptiTaq (EURx) (Lane, 1991). Thermal cycling was as follows: initial denaturation at 95°C for 10 min, denaturation at 95°C for 1 min, annealing at 56°C for 1 min, extension at 72°C for 1 min for a total of 30 cycles, followed by a final extension at 72°C for 10 min and cooling at 4°C for 5 min. PCR products (∼1500 bp) were purified using the GeneMATRIX Agarose-Out DNA Purification Kit (EURx) and screened by RFLP analysis using *Hae*III (Takara Bio) and *Alu*I (FastDigest, Thermo Scientific) endonucleases (Menendez-Vega et al., 2007). The 16S rRNA genes of strains displaying distinct RFLP profiles were sequenced by the Sanger method. The partial (∼800 bp) sequences obtained were compared to those deposited in the 16S RefSeq NCBI database using BLAST algorithm^2^.

### Arsenic Tolerance and Arsenic Resistance Genes in Isolated Bacteria

The sensitivity of the strains collected from the Nitrastur site to arsenic was tested by an agar-dilution method, in the above described 1:10 TSA medium (control), and TSA medium with As(III) (sodium arsenite, Fluka) at concentrations of 2, 5, 10, 15, and 20 mM, or As(V) (sodium arsenate dibasic heptahydrate, Sigma) at concentrations of 1, 20, 50, and 100 mM. The plates were incubated at 30°C for five days and observed for bacterial growth. The presence of genetic determinants of arsenic resistance was analyzed by PCR amplification from the bacterial DNA of *arsB*, *arrA*, *aioA* and two types of *acr3* genes (Achour et al., 2007; Ben Fekih et al., 2018). OptiTaq PCR Master Mix (EURx), using different primers and conditions (Supplementary Material SM1), was used for the analysis.

### Bacterial Community Analyses

Total DNA was isolated from the above-mentioned concentrated groundwater samples. To this end, 1.5 mL of each sample was centrifuged at 11,600 *g*, resuspended in 20 μL water and processed with the DNeasy PowerSoil Pro Kit (Qiagen). The bacterial diversity of the samples before and after addition of nZVI was determined by genetic fingerprinting analysis through ARISA (Automated Ribosomal Intergenic Spacer Analysis). The intergenic spacer region between 16S and 23S bacterial rRNA genes was amplified by PCR using the ITSF and ITSReub primers (Cardinale et al., 2004). Fragment analysis was performed at the Scientific and Technical Services of the University of Oviedo on an ABI PRISM 3130xl Genetic Analyzer (Thermo Fisher Scientific) using 500 ROX internal size standard (Thermo Fisher Scientific). The resulting electropherograms were interpreted with Peak Scanner v.1.0 software (Applied Biosystems). Operational taxonomic unit (OTU) tables were generated using a custom R binning script (Ramette, 2009) that implemented a shifting window binning strategy (Hewson and Fuhrman, 2006). Fragments between 160 and 1000 bp were assigned to bins of 2 ± 0.9 bp, with a minimum Relative Fluorescence Intensity (RFI) cut-off value of 0.1%. The Shannon-Wiener diversity index, Pielou’s Evenness and Inverse Simpson index (1/D) were calculated to characterize *alpha* diversity. Differences between bacterial communities were analyzed by calculating the Bray-Curtis dissimilarity matrix and performing analysis of similarities (Clarke, 1993) and Non-Metric Multi-Dimensional Scaling (NMDS) ordination. Indicator species analysis (De Cáceres et al., 2010) was also carried out.

Microbial diversity was studied at higher resolution and depth using Illumina sequencing. The V4-V5 hypervariable region of the 16S rRNA gene was amplified by PCR using primers 515F (5′-GTGYCAGCMGCCGCGGTA-3′) and 928R (5′-CCCCGYCAATTCMTTTRAGT-3′) with added Illumina-specific adapters (Wang and Qian, 2009; Parada et al., 2016). PCR reactions were carried out using AmpliTaq Gold 360 Master Mix, with 10 min of initial denaturation of DNA at 95°C, followed by 30 cycles of 30 s denaturation at 95°C, 30 s annealing at 60°C, 30 s extension at 72°C and a final extension at 72°C for 7 min. Illumina MiSeq 2 × 300 sequencing was performed by Macrogen (Seoul, South Korea). Sequencing results were analyzed with QIIME2 (version 2018.11). The DADA2 algorithm (Callahan et al., 2016) was used to remove primer sequences from the reads, filter them by quality, dereplicate them, and remove singletons and chimeric sequences. Taxonomic classification of the obtained Amplicon Sequence Variants (ASVs) was performed against the Silva 128 database^3^, with additional input from classification against the NCBI database (Sayers et al., 2011) using BLAST Read and Operational Taxonomic Unit Consensus Classifier (BROCC) software (Dollive et al., 2012). Abundance-based coverage estimator (ACE), Faith’s phylogenetic diversity, Lladser’s point estimate, Effective number of species/probability of intra- or inter-specific encounter (ENSPIE) metrics, the Shannon-Wiener index and Pielou’s *J* (evenness) were calculated in QIIME2 to characterize the diversity of each sample.

### Detection of the Dissimilatory Sulfite Reductase Gene

The presence of the dissimilatory sulfite reductase gene *dsr* (Odom and Peck, 1984) was detected by PCR amplification with the primers DSR1F (5′ACSCAYTGGAAGCACG 3′) and DSR4R (5′ GTGTAGCAGTTACCGCA 3′) (Wagner et al., 1998). The reaction conditions were as follows: initial denaturation at 94°C for 10 min, followed by 30 cycles of 30 s denaturation at 94°C, 54 s annealing at 54°C, a 2-min extension at 72°C and a final extension at 72°C for 7 min.

## Results and Discussion

### Monitoring of Arsenic and Other Physico-Chemical Parameters

**FIGURE 3:**
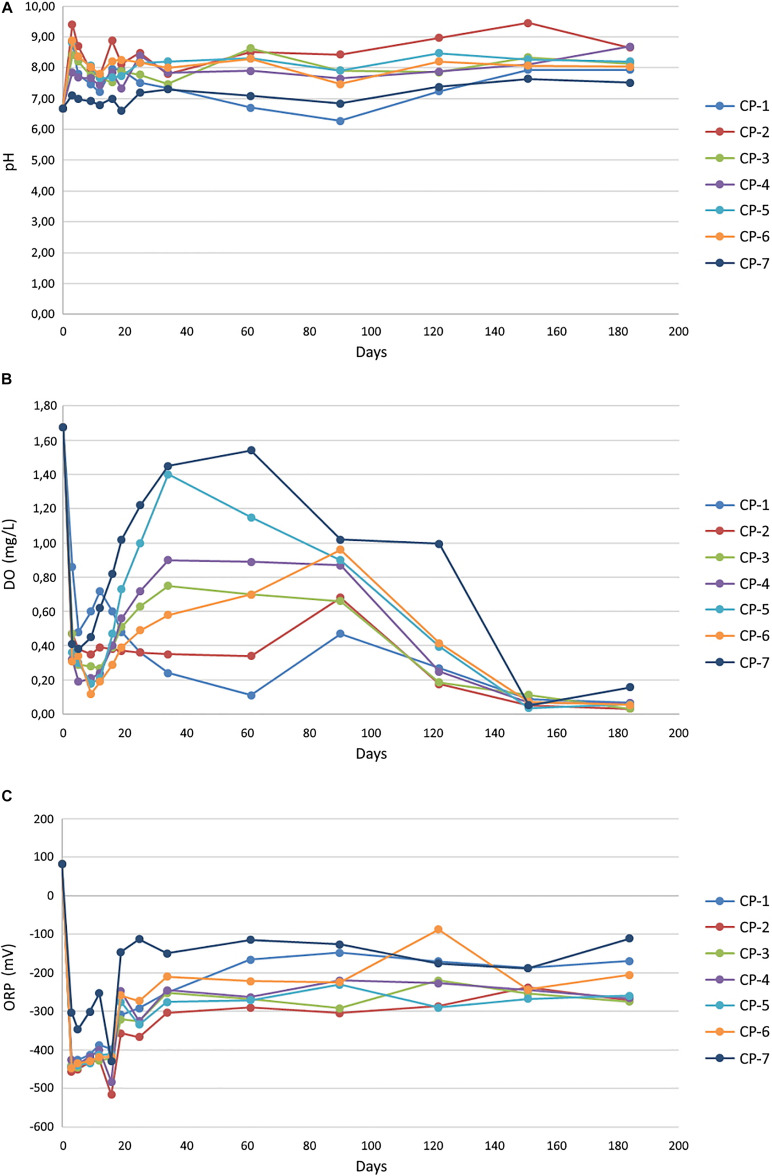
Groundwater pH **(A)**, oxygen concentration **(B)** and oxidation-reduction potential (ORP) **(C)**, in the different wells of the soil, before (0) and after the injection of nZVI nanoparticles. The values obtained for individual wells correspond to the average of three measurements with a standard error < 5%.

The concentration of arsenic and six metals (Cd, Cu, Hg, Ni, Pb, and Zn) in the Nitrastur groundwater is shown in Table 1. Given the groundwater environmental conditions at the site, arsenic is likely to be predominantly in As(V) form. nZVI injection modified the groundwater pH and altered the ORP (Figure 3). Before the addition of the nanoparticles, the pH was slightly acidic (6.6). However, one week after the injection of nZVI, the pH increased to 8.5 in wells CP-1 to CP-7, possibly due to Fe^0^ oxidation (see below) and stabilized at around 8.0 after day 20 and until the end of the experiment (184 days) (Figure 3A).

**TABLE 1:** Basic physico-chemical parameters of Nitrastur groundwater before nZVI application.

**Parameter**	**Average**
**T^a^ (°C)**	13.77
**pH**	6.6
**DO (mgL^–1^)**	1.68
**ORP (± mV)**	96.44
**EC (μScm^–1^)**	1220.16
***Total element concentration* (μ*gL*^–^*^1^)***	
**As**	1796.14
**Pb**	16.31
**Zn**	198.80
**Cu**	42.84
**Cd**	1.53
**Ni**	11.82
**Hg**	0.02
**Nitrate (mgL^–1^)**	1.092
**Orthophosphate (mgL^–1^)**	0.42
**Sulfate (mgL^–1^)**	460.00
**DOC (mgL^–1^)**	2.12

*Temperature (T^*a*^), dissolved oxygen (DO), oxidation-reduction potential (ORP), electrical conductivity (EC), and dissolved organic concentration (DOC). Results correspond to the average of three measurements with a standard error < 5%.*

The oxygen levels in the groundwater before the addition of nZVI (around 1.7 mgL^–1^) corresponded to a microaerophilic environment, and its redox potential (ORP) at the beginning of the analysis was to some extent oxidative (96.44 mV). The application of nZVI caused a notable decrease in the oxygen content and ORP (around −425.8 mV after 3 days), driven by the oxidation of iron, thus creating a strongly reducing environment (Figures 3B,C). Although not observed in all wells, the oxygen content dropped to practically anaerobic conditions after around 90 days. ORP fluctuated in every well during the experiment, but never resulted in oxidative conditions (−222.7 mV at 184 days). In other laboratory experiments at the microcosm scale, the addition of ZVI nanoparticles to river water also led to a rapid decrease in ORP and dissolved oxygen (Barnes et al., 2010). In that study, ORP returned to the initial values after three days of treatment, whereas recovery of the initial oxygen concentrations was achieved only at the end of the experiment (36 days). Therefore, although the initial effect was the same as that observed in our work, the differences in the evolution of these parameters are attributable entirely to distinct environmental conditions in the two cases, which also have a great influence on other results, as shown later.

Nanoscale zero-valent iron is highly susceptible to corrosion in aqueous medium, being rapidly oxidized to Fe(II) by dissolved oxygen and subsequently, and more slowly, to Fe(III) (Pullin et al., 2017). In this regard, the addition of nZVI to aqueous systems usually increases the pH, due to the generation of OH^–^ from the reduction of water by Fe^0^ (Sun et al., 2006; O’Carroll et al., 2013). We observed a moderate increase in the pH of the groundwater after the application of nZVI (Figure 3A). A rapid increase in pH to 8.6 one day after the application of nZVI has also reported in an *in situ* pilot-scale remediation of a chromium-contaminated site (Němeček et al., 2014). The magnitude of the increase in that study was greater than at the Nitrastur site since the pH of the chromium-contaminated groundwater before the treatment was slightly acidic rather than alkaline. Furthermore, in the microcosm experiments with river water mentioned above (Barnes et al., 2010), the addition of nZVI slightly reduced the pH during the 36 days of the experiment. The authors attributed this decrease to the microbial production of organic acids since in sterile water the pH increased in the presence of nanoparticles. Therefore, the variation in pH is conditioned by the water chemistry (including the possible presence of organic acids) at the sites analyzed.

**FIGURE 4:**
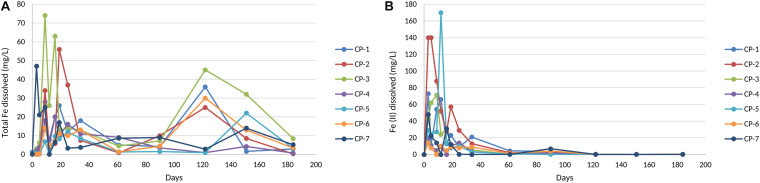
Total Fe **(A)** and Fe (II) dissolved **(B)** in groundwater from the nZVI injection until the end of the experiment. The values shown of each well correspond to the average of three measurements with standard error < 5%.

nZVI treatment of the groundwater led to an increase in the concentration of total dissolved iron during the first 20–30 days of the treatment (Figure 4A), with mean values of around 28 mgL^–1^ at around 10 days (74 mgL^–1^ in some of the wells, as in CP-3). After this, a marked decrease in this parameter was observed during the next 80 days, followed by further fluctuations until the end of the study. Consistent with the oxidative processes mentioned, Fe(II) concentration increased markedly during the first month of treatment due to the nZVI-water reaction (Figure 4B). This kinetics and the subsequent disappearance of Fe(II) points to its further oxidation to Fe(III).

**FIGURE 5:**
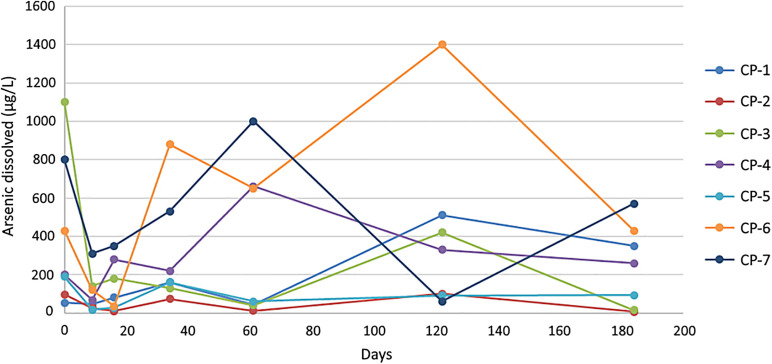
Dissolved arsenic concentration (μgL^–1^) in groundwater at the different injection wells during remediation. The values shown for the individual wells correspond to the average of three measurements with a standard error < 5%.

The application of nZVI to the groundwater led to a dramatic decrease (75% on average in all wells) in the concentration of arsenic during the first 10 days. Nevertheless, the concentrations of this metalloid increased again and fluctuated in most of the wells after the first month and until the end of the six-month experiment (Figure 5). Conceivably, arsenic is adsorbed/co-precipitated onto the iron oxides created by the oxidation of nZVI, a process that would drive its immobilization and therefore the initial decrease observed (O’Carroll et al., 2013; Jiang et al., 2018). Several factors could explain the increase in arsenic in groundwater after the first month. On one hand, the aging/oxidation of nZVI leads to a decrease in the content of Fe^0^ and the reducing equivalents available and thus the adsorption of metals (O’Carroll et al., 2013). On the other hand, the aggregation and mobility of nZVI are influenced by the ionic strength of the water and its composition (Keane, 2009). Such aggregation lowers the available surface of the nZVI nanoparticles and therefore their effectiveness, and also reduces their mobility. If the transport of the nanoparticles is not efficient, they will oxidize to form Fe(II), Fe(III) and/or iron oxides and will disappear from the water by settling (O’Carroll et al., 2013).

Confocal laser scanning microscope images of some of the groundwater samples three days after the addition of nZVI show a certain degree of nanoparticle aggregation, in some cases greater than 150 μm (Supplementary Material SM2), thus, the effects of such phenomenon on the efficiency of the groundwater remediation need to be further analyzed. An increase in groundwater pH (Figure 3A) has been reported to affect transport, mobility and adsorption of contaminants (Zhang and Elliott, 2006), including impairment of the removal of both species of arsenic due to electrostatic repulsion (Park et al., 2009). However, a significant effect of the nanoparticles on the adsorption of As(V) in the groundwater of Nitrastur is unlikely since the highest pH value reached was well below the values that induce a marked decrease of nZVI surface potential and would therefore greatly hinder the adsorption of this form of arsenic (Park et al., 2009; Wu C. et al., 2017). On the other hand, an increase in the pH of groundwater alters the proportion of corrosion products of nZVI, such as maghemite, which has a higher adsorption affinity toward As(V) (Wu C. et al., 2017).

The *in situ* arsenic remediation work at Nitrastur is subject to complex variables that influence the effectiveness of the process. Also, it should be considered that the groundwater environment is not static as the study area receives water from other sources, thereby producing a continuous flow of arsenic. Furthermore, the microbial activities related to the metabolism of arsenic, iron and possibly nitrogen and sulfur are likely to also play a relevant direct or indirect role in the observed fluctuations.

### Isolation, Identification and Characterization of Cultured Bacteria

Microbiological data was collected in the four wells nearest to the injection points (CP-2, CP-4, CP-5, and CP-6) (Figure 1). The analysis of cultured bacteria provided useful information about their biochemical characteristics, behavior during treatment and possible biotechnological applications.

**FIGURE 6:**
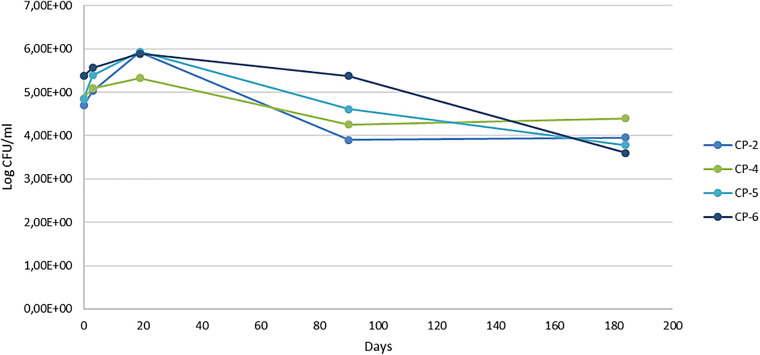
Viable bacterial counts (colony forming units, CFU) per milliliter at different sampling times of the nZVI treatment (0, 3, 19, 90, and 184 days).

Cultured bacterial counts showed an increase in viable cells in the first 18 days of nZVI treatment, followed by a continuous decrease, with numbers being lower at the end of sampling (around 20% for the four wells analyzed) than at the beginning (Figure 6). An initial increase in both microbial number and Fe(II) was also described in the aforementioned laboratory experiments on river water at microcosm scale (Barnes et al., 2010). That study also reported an increase in viable bacteria the first day after nZVI addition accompanied by a decrease in the concentration of oxygen and ORP (which recovered later, returning to initial levels). The authors linked the initial increase in the number of bacteria to the presence of Fe(II), which stimulated the growth of Fe(II)^–^-oxidizing bacteria in the microaerophilic conditions created. The subsequent decrease observed in the number of cultured bacteria was attributed to the disappearance of Fe(II). In our case, however, the restructuring of the bacterial population composition that accompanied the establishment of full anaerobic conditions (see below) is probably the most relevant factor.

It is difficult to generalize the results of this study to similar ones in this regard. In the *in situ* remediation of Cr(VI)-contaminated groundwater with nZVI, no inhibition of the density of cultured psychrophilic bacteria was observed nor of the specific phospholipid fatty acid concentrations of the bacteria present (Němeček et al., 2014). Another long-term field study, which combined nZVI with electrokinetic treatment for the remediation of chlorinated ethenes (Czinnerová et al., 2020), reported a temporary negative impact on total biomass of indigenous bacteria in the well near the cathode but not in the well near the anode. This finding was attributed to a dilution/displacement effect of the nZVI suspension or the high doses of nZVI, although the microbial population was restored within 15 days. Other studies on the effects of nZVI at laboratory scale (microcosm), on samples of an aquifer contaminated with trichloroethylene, report a negative impact on indigenous microbial communities, as measured by DNA qPCR analysis, after 8 months of treatment (Kirschling et al., 2010).

### Bacterial Identification

30 morphologically distinct strains were isolated, grown in TSA medium and identified (Supplementary Material SM3). Half of these strains was isolated before addition of nZVI, while the rest were isolated from groundwater sampled at different times during the course of the experiment. Overall, the predominant phylum was *Proteobacteria* (13 strains, with representatives from the *alpha*-, *beta*-, and *gamma*-classes), followed by *Actinobacteria* (11 strains), *Firmicutes* (4 strains) and *Bacteroidetes* (2 strains). Most of the phyla of isolated cultured bacteria (*Proteobacteria* and *Actinobacteria*) seem to predominate among bacteria that live in oligotrophic environments (Huang and Shen, 2016), as was the case of the groundwater at the Nitrastur site. Such environments determine the biology of the microorganisms present, as we will discuss later.

There were notable changes in cultured bacteria after application of nZVI. Before the application, these were mostly (about 71%) gram-negative, and afterward this proportion dropped to 38% and was accompanied by a change in the taxonomic composition. *Proteobacteria* were the most abundant before the nZVI treatment, while representatives of *Actinobacteria* were the most numerous after the treatment. Most of the bacteria isolated before the application of nZVI were also detected at some of the sampling times after addition of the nanoparticles, and eight of them (five gram-negative) were detected at the end of the experiment (184 days). In a pilot-scale *in situ* remediation of a chromium-contaminated site with nZVI (Němeček et al., 2014), the authors did not detect significant changes in the relative abundance—determined by total phospholipid fatty-acid analysis—of the bacterial groups in the groundwater; however, they reported stimulation of gram-positive bacterial growth in the soil, which they attributed to the increase in iron after addition of the nanoparticles. Our data also suggests that the change in the taxonomic composition of the groundwater bacteria after addition of nZVI may be due to the altered environmental conditions and not due to the specific direct effects of the nanoparticles on the gram-positive or gram-negative bacterial groups. This notion is supported by the data on bacterial viability in the water samples, as shown by confocal laser scanning microscopy after staining with the PI and SYTO 9. Three days after the addition of nZVI, the analysis revealed that most of the bacteria visible under the microscope were viable (Supplementary Material SM2).

A particularly resilient bacterial strain was *Acidovorax* (*beta*-*Proteobacteria* class), which was present in all wells during most of the time points analyzed (Supplementary Material SM3). The genus *Acidovorax* has various abilities related to arsenic and nitrogen cycles (genetic determinants for arsenite oxidation, denitrification, dissimilatory nitrate reduction to ammonia and ammonification (Huang et al., 2012; Suhadolnik et al., 2017 and references therein). Arsenic adsorbs to natural Fe(III) (oxyhydr)oxides regardless of whether they were derived from abiotic or biotic oxidation of Fe(II) (Hohmann et al., 2011). Anaerobic respiration of nitrate coupled to Fe(II) oxidation (nitrate-dependent iron oxidation, NDFO) has been long known (Carlson et al., 2013), and various mixotrophic *Acidovorax* species have this capacity (Carlson et al., 2013 and references therein). Nitrate-dependent *Acidovorax* species generate biogenic Fe(III) (oxyhydr)oxides, which show stronger adsorbency to As(V) than to As(III) (Hohmann et al., 2011; Sowers et al., 2017). Given that nitrate reduction is associated wih the anoxic oxidation of arsenite (Sun et al., 2008), the simultaneous microbial oxidation of Fe(II) and As(III) facilitated by nitrate may be a key process leading to the formation of ferric oxide and As(V) particles, resulting in immobilized arsenic in the form of As(V) adsorbed on biogenic Fe(III) (oxyhydr)oxides, with reduced mobility and toxicity (Hu et al., 2015). nZVI can also reduce nitrate (Yang and Lee, 2005), and nitrate slows the aging of this nanomaterial (Reinsch et al., 2010). Thus, it is feasible that the interactions of *Acidovorax* (and other bacteria present in all the wells, such as *Hydrogenophaga*, which remained throughout the nZVI treatment) with As and nitrate, which are linked to the geochemical conditions stimulated by the addition of nZVI, could have a relevant role in the biogeochemical cycles during the remediation of the Nitrastur site. The genus *Hydrogenophaga* (*beta*-*Proteobacteria* class), a class of oligotrophic bacteria that can develop chemorganotrophically or chemolithoautotrophically, performs growth-linked heterotrophic As(III) oxidation. This genus is abundant in natural water, wastewater and other environments, where it can oxidize Fe(II), hydrogen and thiosulfate, grow in ferrous minerals such as FeS and FeCO_3_ and perform hydrogenotrophic denitrification (Jewell et al., 2017; Luo et al., 2018 and references therein).

Also, *Sphingopyxis* (*alpha*-*Proteobacteria* class), which was isolated from the groundwater, is a genus that comprises versatile bacteria that are present in a wide variety of environments, including seawater, wastewater treatment plants, and hydrocarbon-contaminated soils. These bacteria harbor genes that code for resistance against arsenic, metals and many efflux pumps, and some species can also use nitrate as a terminal electron acceptor in anaerobic respiration (Verma et al., 2020). *Microbacterium* is a genus of Actinobacteria that includes species with a high capacity for interaction with and resistance to arsenic and other metals (Kaushik et al., 2012). A strain related to *M. oxydans* was isolated from the nZVI-treated groundwater of the Nitrastur site. Several strains of this species can adsorb sodium arsenite (Aksornchu et al., 2008) and remove lead, nickel and copper (Heidari et al., 2020). *Pseudomonas* species, on the other hand, can degrade aromatic compounds, and the derived intermediates further serve as electron donors to reduce iron, nitrate and arsenate (Jiang et al., 2019). Denitrification and arsenic resistance are also common features of the genus *Bacillus* (Verbaendert et al., 2011; Ben Fekih et al., 2018). Finally, members of the genus *Aminobacter* (*alpha*-*Proteobacteria* class) appear to be widely distributed in terrestrial environments (Sørensen et al., 2007 and references therein) and show high metabolic versatility, although they appear to be unable to carry out denitrification (Urakami et al., 1992).

### Arsenic Tolerance and Presence of Arsenic Resistance Genes in the Isolated Bacteria

The 30 bacteria isolated and identified were grown in TSA medium with As(III) and As(V) to test their tolerance. Most of them grew in the presence of the minimum concentration of As(III), although eight strains (those from *Microbacterium* sp., *Microbacterium oxydans*, *Microcella putealis, Sphingopyxis* sp., *Planomicrobium okeanokoites, Pseudomonas parafulva*, *Bacillus cereus* and *Pseudomonas brenneri*) were tolerant to concentrations of As(III) of 5 mM or higher (Supplementary Material SM4). *Microbacterium* species are the dominant bacteria reported in other heavily contaminated sites and are conceivably involved in the biochemical cycles of arsenic (Kaushik et al., 2012). Four of the isolated strains (*Massilia* sp., *Acinetobacter lwoffii*, *Pseudomonas peli* and *Ideonella* sp.) did not tolerate even the minimum As(III) concentration, and the last two did not grow at any As(V) concentration. Most of the bacteria tested, including the seven As(III)-resistant strains mentioned above, were tolerant to 100 mM As(V). The data suggest an evolutionary adaptation of the bacterial community over time to contamination with arsenic, which would bring about the presence of resistance genes to this metalloid element (Ben Fekih et al., 2018). We performed PCR amplification of the bacterial DNA of the following genes involved in four strategies used to respond to arsenic toxicity: (a) *arsB*, involved in the expulsion of As(III) after As(V) detoxifying reduction, the latter driven by the *arsC*; (b) *arrA*, responsible for the respiratory reduction of As(V) to As(III); (c) *aioA*, which oxidizes As(III) to a less toxic As(V) product; and (d) *acr3.1* and *acr3.2*, whose products are also a pump that expels As(III) from cells.

Amplification of one or more genes conceivably related to arsenic resistance was observed in all the bacteria, except five (Supplementary Material SM4). The genes of interest were found mainly in the *Actinobacteria* class and the different classes of *Proteobacteria*, except *aioA*, which encodes As(III) oxidase and was most frequently detected in the latter. The most detected gene was *acr3*, responsible for the expulsion of As(III). This gene was amplified in half of the 30 bacteria assayed, followed by the As(III) oxidation gene *aio*, detected in 12 bacteria. We also observed that *acr3* predominated in *Actinobacteria* and *alpha*-*Proteobacteria* and was more abundant than *arsB* in the cultured bacteria studied (Supplementary Material SM4). In a forest soil analysis (Achour et al., 2007), the presence of genes related to *arsB* or *acr3* was greater than 70% in the bacterial isolates, and some individual strains contained both; furthermore, like our study, *acr3* predominated in *Actinobacteria* and *alpha*-*Proteobacteria* and was more abundant than *arsB*. In the study of the genome of 2,500 bacterial strains (Yang et al., 2015), *acr3* and *arsB* were also the most prevalent. A recent global study of genes related to arsenic in soil microbiomes has shown that, in cultured bacteria, *acr3* and *arsC* (indicative of arsenic reduction and extrusion detoxification pathways) are predominant in the soils (*arsC* being generally the most abundant), and genes related to arsenic metabolism (such as oxidative *aioA*) are much less prevalent (Dunivin et al., 2019). However, our data revealed that *aioA* was present in at least 36% of the bacteria analyzed, although this discrepancy may be due to the relatively low number of strains studied rather than to the nature of the specific site.

The distribution of the two families of arsenic efflux pumps, *acr3.1* and *acr3.2*, differed in the analyzed cultured bacteria. *acr3.1* predominated in *Actinobacteria* and *acr3*.2 in *Proteobacteria*, which is a similar general distribution to that reported in forest soils (Achour et al., 2007) and that reported for *acr*3.2 in soils (Dunivin et al., 2019). *arrA* is highly conserved (Messens and Silver, 2006), although we detected it in only 9 of the 30 bacteria analyzed. It has been proposed that this gene, which is associated with arsenate respiration, is present mainly in bacteria that live in highly polluted environments (Desoeuvre et al., 2016). In contrast, we detected *arsB* and *arrA*, involved in the detoxification and respiratory reduction of As(V), in the genome of three bacteria (Supplementary Material SM4). Something similar was found with *acr3.1* and *acr*3.2, which were detected in the same genome of two actinobacteria (*Arthrobacter chlorophenolicus* and *Dermacoccus nishinomiyaensis*) and one *Proteobacteria* (*Sphingobium limnetium*) (Supplementary Material SM4). This coexistence in the same chromosome has been described previously (Cai et al., 2009), and indicates that the presence of these genes with redundant function in the same bacteria is not mutually exclusive.

Soil bacterial strains with both an arsenite oxidation gene and an arsenite transporter (*acr3* or *arsB*) have been postulated to show greater resistance to arsenite (Cai et al., 2009). This pattern did not appear in our results as we did not detect a relationship between the levels of resistance to As(III) or As(V) and the absence or presence of the different genes tested. The strains of *Pseudomonas peli* and *Ideonella* sp. that did not show growth in the minimal concentrations of As(III) and As(V) carried *acr3* (both strains), *arrA* (*P. peli*), and *aio* (*Ideonella* sp.). Therefore, these genes may not be functional under the conditions tested (Lescure et al., 2019). Genome sequencing and expression analysis experiments in these strains (Dunivin et al., 2019) are required to address this contradictory result. Another conflictive aspect of our findings is the apparent absence of related genes in five strains which present high levels of resistance to arsenic, especially to As(V) (Supplementary Material SM4). This observation could be explained by the presence of alternative resistance genes, like those related to arsenite methylation, which are relevant in aquatic ecosystems, and/or by the presence of genes with highly divergent DNA sequences (Achour et al., 2007; Desoeuvre et al., 2016).

### Bacterial Community Diversity and Structure

Despite its usefulness, the information obtained with cultured bacteria is limited because these studies underestimate the true metabolic potential of the population as a whole in relation to arsenic, as well as its response to treatment with nZVI. Thus, we used molecular fingerprinting methods to obtain a complete profile of the diversity of the bacterial community in the groundwater during the treatment with nZVI. In this regard, ARISA (Automated Ribosomal Intergenic Spacer Analysis) was used to analyze the ecosystem dynamics, and Illumina sequencing of 16S ribosomal RNA gene regions for taxonomic identification and characterization of the bacterial communities.

### ARISA Analysis

**FIGURE 7:**
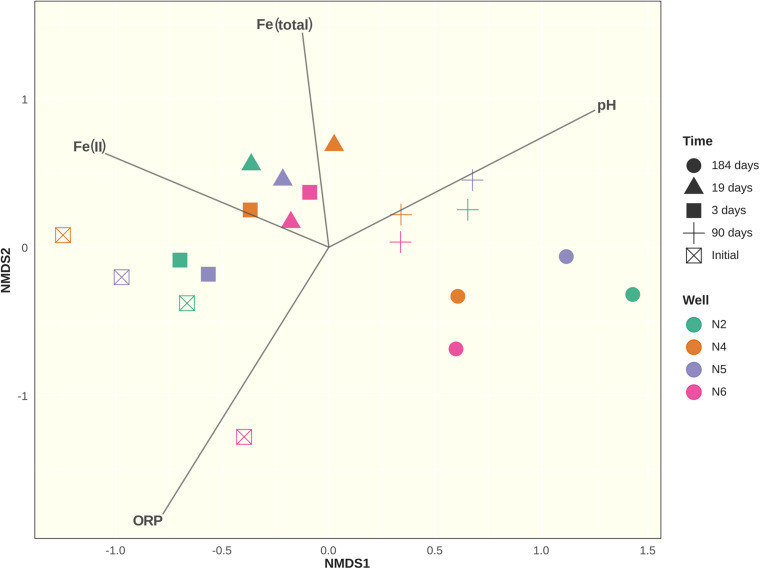
Non-metric Multidimensional Scaling (NMDS) ordination plot of Bray-Curtis community dissimilarities based on bacterial ARISA profiles with added ordination for environmental variables: Fe(II) concentration in μgL^–1^; total iron content in mgL^–1^; redox potential (ROP) and pH. Axes represent arbitrary distance.

DNA was extracted from samples taken from wells CP-2, CP-4, CP-5, and CP-6 before the beginning of the experiment, and at 3, 19, 90, and 184 days after application of the nanoparticles. A total of 20 samples were thus processed. PCR reactions were performed in duplicate using ITSF primers labeled with different fluorochromes. Peaks identified by the Peak Scanner software were binned into 256 operational taxonomic units (OTUs). After positive results of the similarity check on the duplicates, they were merged by averaging RFI values for each OTU. Differences between sample groups were observed on the NMDS plot (Figure 7). At days 3 and 19 after application of the nanoparticles, bacterial communities became grouped together on the NMDS plot. This period also corresponded to the maximum concentrations of Fe(II) nanoparticles and increased total iron content due to their oxidation, which is reflected by the change in the ORP. Microbial communities 90 days after the application of nanoparticles differed from those present at days 3 and 19, as well as from communities inhabiting groundwater sediments before the treatment. This tendency continued into the experiment, with microbial communities on day 184 diverging even further both from the original populations and from the communities observed in the first weeks after the application of nanoparticles.

Analysis of similarities (ANOSIM) using the Bray-Curtis dissimilarity matrix revealed no differences between microbial communities when grouped by sampling well (*R* = 0.04367; *p* = 0.252). However, differences between groups were detected when grouped by sampling time (*R* = 0.5654; *p* = 0.001). Indicator species analysis showed that of the 256 OTUs, 41 were differentially abundant and associated with sampling times (p < 0.05). The most abundant of these OTUs are shown in Supplementary Material SM5. While ARISA does not have the depth, resolution nor taxonomic identification capacity of high-throughput sequencing methods such as Illumina or pyrosequencing, β-diversity estimates derived from ARISA data were found to be mostly consistent with these methods (Gobet et al., 2014; van Dorst et al., 2014). In our study, the Bray-Curtis dissimilarity matrix calculated from a smaller Illumina dataset had high similarity to its counterpart generated from ARISA data (Mantel test: *r* = 0.7635, *p* = 0.006; Procrustes analysis: *m^2^* = 0.24, *r* = 0.8718, *p* = 0.007; in both cases, *p*-value calculated after 1000 permutations).

**FIGURE 8:**
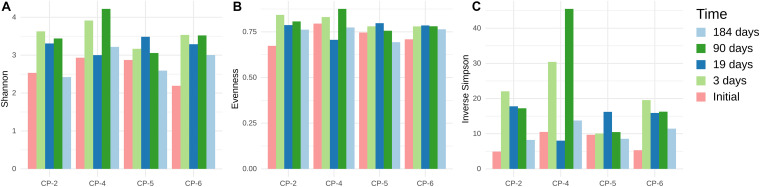
*Alpha*-diversity indices of microbial communities in groundwater samples from CP-2, CP-4, CP-5 and CP-6 monitoring wells, before the treatment (Initial) and at different times after the addition of nZVI, calculated from ARISA data. **(A)** Shannon-Wiener index; **(B)** Pielou’s Evenness; **(C)** Inverse Simpson index (1/D).

In the first 19 days after application of the nanoparticles, the composition of the bacterial communities in all the samples showed a dramatic alteration, with populations in wells CP-2 and CP-5 changing at a slower pace. After 90 days of treatment, the communities in all the wells were relatively similar. Despite further minor changes, this similarity persisted. Interestingly, *alpha*-diversity indices (Figure 8) varied depending on the well and sampling time but with no clear pattern. These observations suggest that, despite pronounced shifts in the community structure after injection of nZVI, the overall level of bacterial diversity was not negatively affected. However, the Illumina data subsequently contradicts this assumption, as discussed below.

### Prokaryotic Community Structure

**FIGURE 9:**
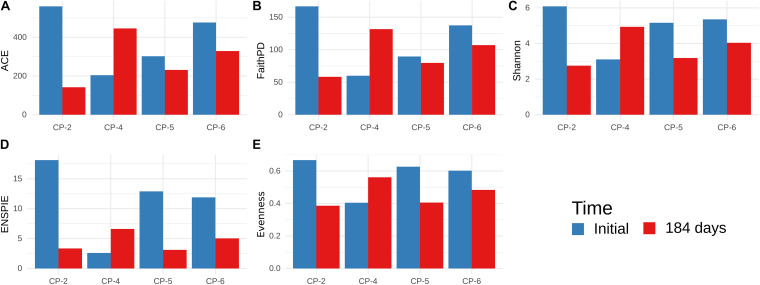
*Alpha*-diversity of microbial communities in groundwater samples from CP-2, CP-4, CP-5, and CP-6 monitoring wells, before the treatment (Initial) and at 184 days after the addition of nZVI, calculated from Illumina data. **(A)** Abundance-based Coverage Estimator; **(B)** Faith’s Phylogenetic Diversity; **(C)** Shannon-Wiener index; **(D)** ENS_PIE_ metrics; **(E)** Evenness (Pielou’s *J*).

Analysis of the data of 16S rDNA Illumina sequencing indicated that almost all the sequences detected in the samples from the four wells belonged to the Domain Bacteria and only 0.157% of the amplicon sequence variants (ASV) were from Archaea. *Alpha*-diversity was studied on a dataset rarefied to the depth of 53850 ASVs (corresponding to the sample with the lowest ASV count). Good’s coverage of reads at that depth was > 0.999 for all samples, and average Lladser’s point estimate was 5.61 × 10^–4^ (σ = 1.5 × 10^–4^), indicating that ASVs with abundances of 10^–4^ or higher were included in the rarefied dataset. Figure 9 summarizes the *alpha*-diversity of the microbial communities in the four monitoring wells at the beginning and the end of the treatment with nZVI.

Before application of nZVI, all the indices were lower in the sample from the CP-4 well compared to those from CP-2, CP-5, and CP-6. However, the diversity in CP-4 increased at the last observed time (184 days), while the higher initial diversity in the other wells was diminished. CP-2 was the most severely affected, with a significant decrease in species richness (ACE and Faith’s PD) and evenness (ENS_PIE_ and Pielou’s *J*), while CP-5 showed reduced evenness with very little impact on species richness. Such different *alpha*-diversity patterns could be attributed to the heterogeneous conditions of the sites (Portell et al., 2018). In contrast to other studies (Gobet et al., 2014; van Dorst et al., 2014), we found that *alpha*-diversity estimates based on ARISA and Illumina datasets were not in agreement, with the Illumina dataset showing that diversity was affected more than initially thought.

**FIGURE 10:**
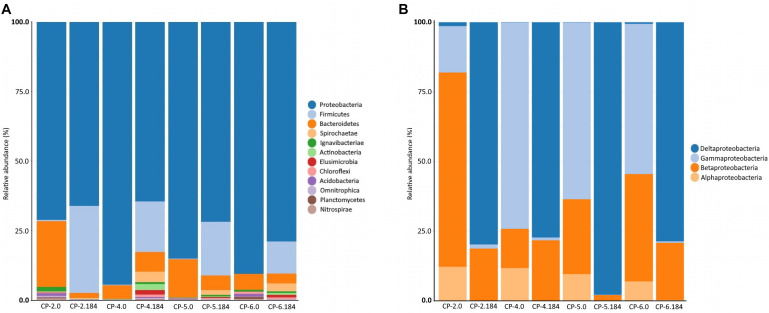
Phyla abundance **(A)** and *Proteobacteria* class abundance **(B)**, in the samples of Nitrastur groundwater from CP-2, CP-4, CP-5 and CP-6 monitoring wells before the treatment (0) and at the end of the treatment (184 days).

In addition to the observed changes in diversity, the composition of the bacterial community was also modified over time by the nZVI treatment. The phylum *Proteobacteria* was dominant in the groundwater before and after the application of nZVI (Figure 10A and Supplementary Material SM6). However, before the application of the nanomaterial, bacteria of the *gamma*-*Proteobacteria* class predominated in three wells (CP-4, CP-5, CP-6), while members of the *beta*-*Proteobacteria* class were the most prevalent in CP-2 (Figure 10B) where bacteria from the phylum *Bacteroidetes* were the second most abundant phylum (between 5.0% and 23.7%). Other groups (phylum *Chloroflexi*, class *Ignavibacteria*, phylum *Acidobacteria* and others) each accounted for less than 1% of total counts. At the end of sampling (184 days after nZVI injection), the abundance of the phylum *Bacteroidetes* decreased dramatically, whereas bacteria from the phylum *Firmicutes* increased notably (11.6% to 31.3% in all four wells) (Figure 10A and Supplementary Material SM6). In addition, the *delta*-*Proteobacteria* class substituted the *gamma*-*Proteobacteria* and *beta*-*Proteobacteria*, while bacteria of the *alpha*-*Proteobacteria* class, which were the third most abundant taxonomic level before the addition of nZVI, practically disappeared (Figure 10B). These changes most likely reflect an adaptive mechanism of the bacterial communities to the new environmental conditions (pH, oxidation-reduction potential, and finally the predominant anaerobic conditions) produced by the treatment. The distribution of the cultured bacteria before and after the addition of nZVI, with a predominance of the phyla *Proteobacteria* and *Actinobacteria*, is conditioned by the capacity to grow in the medium used, and therefore does not coincide with the distribution obtained from the direct analysis of the populations using molecular techniques (which also shows that the bacteria of the phylum *Actinobacteria* do not predominate in the Nitrastur groundwater). However, an increase in cultured bacteria of the phylum *Firmicutes* was observed after the nZVI treatment, although represented only by genera capable of growing in aerobic conditions (*Bacillus* and *Planomicrobium*).

Considering the results globally, the taxonomic distribution in the groundwater of Nitrastur is repeated to some extent in other environments contaminated with arsenic, such as shallow waters, groundwater, aquifers and freshwater sediments (Lin et al., 2012; Suhadolnik et al., 2017; Cavalca et al., 2019; Jiang et al., 2019). However, a more obvious difference is that the phylum *Firmicutes* has a greater abundance in these environments than in the Nitrastur groundwater (Bertin et al., 2011; Escudero et al., 2013; Hassan et al., 2015; Wang et al., 2016, Suhadolnik et al., 2017; Jiang et al., 2019). This observation could be attributed to the different environmental conditions, which, in the case of Nitrastur, are more favorable for members of the phylum *Firmicutes* after the addition of nZVI (see below).

**FIGURE 11:**
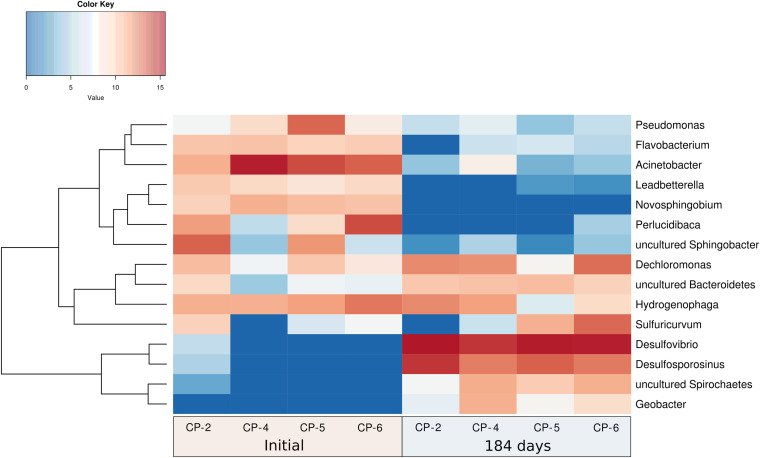
Hierarchical cluster analysis (*Y*-axis), and heatmap showing the relative abundances of the most abundant genera identified in the Nitrastur groundwater samples from wells CP-2, CP-4, CP-5 and CP-6, before (0) and at the end of the experiment (184 days).

At the genus level, differences in both bacterial composition and abundance were also observed in the groundwater samples (Figure 11 and Supplementary Material SM7). Before the addition of nZVI, *Acinetobacter* (41%), *Perlucidibaca* (18%), uncultured *Sphingobacteria* (13%), *Hydrogenophaga* (10%), *Pseudomonas* (7%), *Novosphingobium* (4%), *Flavobacterium* (3.4%), *Dechloromonas* (2.8%) and *Leadbetterella* (2.4%) were the most abundant populations in the four wells. Other genera such as *Sulfuricurvum* and uncultured *Bacteroidetes* were also detected, albeit in lower proportions. The taxonomic structure of the most abundant genera followed the changes observed at the high taxonomic phyla levels (Supplementary Material SM7). Similar to our study in Nitrastur, *Acinetobacter* were found to be the most abundant bacteria in groundwater contaminated with arsenic and rich in Fe(III) and As(V)-reducing bacteria (Xiu et al., 2020). *Acinetobacter* species are widespread in soils and waters affected by natural or anthropogenic arsenic and/or organic pollution, where they are involved in the oxidation of As(III) and can use As(V) for respiration under anoxic conditions (Achour et al., 2007; Cai et al., 2009; Ghosh and Sar, 2013). It has been proposed that these bacteria mobilize arsenic through the reductive dissolution of mineral groups of Fe(III)/As(V), thereby also suggesting that the oxidation of ammonia coupled to the reduction Fe(III) (again pointing to the participation of nitrogen cycle intermediaries in this process) in these environments also contributes to arsenic enrichment in reducing aquifers (Xiu et al., 2020). *Perlucidibaca* is a chemoheterotrophic, facultative aerobic and weakly nitrate-reducing freshwater bacterium associated with hydrocarbon degradation capacity (Song et al., 2008; Lofthus et al., 2020).

Also isolated in culture, *Hydrogenophaga* (*beta*-*Proteobacteria*) is a versatile heterotrophic and chemolithoautotrophic bacterium (with H_2_, CO, and S species, such as thiosulfate, as electron donors) (see above) that was present before and during the entire nZVI treatment. *Novosphingobium* is broadly distributed in water and soil and can degrade a wide variety of organic pollutants and, in some cases, can also reduce nitrates (Kumar et al., 2020). *Flavobacterium* includes species isolated from sediments with a high arsenic concentration (Ao et al., 2014), and *Leadbetterella* is a strictly aerobic bacterial genus (Weon et al., 2005). Similar culture-independent 16S rRNA gene-based studies have shown the presence of *Acidovorax*, *Hydrogenophaga*, *Dechloromonas*, *Sphingobium*, and *Novosphingobium*, in addition to *Geobacter* and sulfate reducers, among other genera, in arsenic-contaminated aquifers of China, Bangladesh and nearby West Bengal (Islam et al., 2004; Hu et al., 2015; Hassan et al., 2015 and references therein; Jiang et al., 2019).

We found that the dominant genera after 184 days of nZVI treatment were *Desulfovibrio* (59%), *Desulfosporosinus* (19%), *Dechloromonas* (7%), *Sulfuricurvum* (5%), *Hydrogenophaga* (3.5%), uncultured *Spirochaetes* (2.6%), uncultured *Bacteroidetes* (2%) and *Geobacter* (2%). This composition was maintained with remarkable similarity in the groundwater samples from all four wells (Figure 11 and Supplementary Material SM7). These genera also reflect the taxonomic change at the level of phyla (decrease of the bacteria from the *Bacteroidetes* phylum, and presence of *delta*-*Proteobacteria* and bacteria from the phylum *Firmicutes*) and indicate adaptation to the new anaerobic conditions caused by the addition of nZVI. This adaptation correlates with the metabolic characteristics of all these bacteria (strict anaerobic metabolism in *Desulfovibrio*, *Desulfosporosinus* and *Geobacter*, or facultative metabolism in *Sulfuricurvum*, *Hydrogenophaga* and *Dechloromonas* – genera which were also present before the treatment), and especially with the significant predominance of *Desulfovibrio* and *Desulfosporosinus*.

*Desulfovibrio*, a genus of bacteria that is widely distributed in oligotrophic and eutrophic environments, reduces sulfate with the concomitant oxidation of substrates such as lactate, ethanol or formate, and hydrogen, which are themselves fermentation products of facultative anaerobes (Baffert et al., 2019). The ability to produce or consume hydrogen provides this bacterium with a relevant role in the interspecific transfer of hydrogen (Baffert et al., 2019). The dissimilatory sulfite reductase gene *dsr* encodes for the key enzyme that catalyzes the six-electron reduction of (bi)sulfite to sulfide, which is the central energy-conserving step of sulfate respiration in the anaerobic sulfate respiration pathway (Odom and Peck, 1984). This gene is present in all known sulfate-reducing microorganisms with a high degree of conservation (Wagner et al., 1998) and, remarkably, in the Nitrastur groundwater, we detected *dsr* in the DNA of all samples at days 90 and 184 of the nZVI treatment. On the other hand, sulfate concentrations in the groundwater before the treatment were on average 460 mgL^–1^ in the wells (Table 1). These levels decreased dramatically soon after the addition of nZVI and reached 125 mgL^–1^ at the end of the treatment (184 days) (data not shown), probably being modulated by bacterial activity.

The sulfate-reducing bacterium *Desulfosporosinus* has been isolated from acid drainage sediments derived from coal mines and other sites, and is capable of metabolizing aromatic compounds. In addition to sulfate, it can use other electron acceptors for growth, such as sulfur, sulfite, thiosulfate, nitrate and arsenate (Liu et al., 2004). *Dechloromonas* is a facultatively anaerobic, denitrifying bacterium that is widely distributed in the environment. It can oxidize H_2_, Fe(II), H_2_S and possibly thiosulfate, and is capable of using aromatic compounds (Chakraborty and Picardal, 2013; Luo et al., 2018). This bacterium, together with *Sulfuricurvum*, was present in the groundwater before the application of nZVI (Figure 11 and Supplementary Material SM7), but its abundance increased significantly after the addition of the nanoparticles. *Sulfuricurvum* is a facultatively anaerobic, chemolithoautotrophic, sulfur-oxidizing bacterial genus that uses sulfide, elemental sulfur, thiosulfate and hydrogen as electron donors and nitrate as electron acceptor under anaerobic conditions (Kodama and Watanabe, 2004). *Geobacter* is a genus comprising heterotrophic bacteria commonly found in groundwater reservoirs contaminated with arsenic (Lovley et al., 2011; Jiang et al., 2019). They can use organic matter (e.g., acetate or lactate) as an electron donor for respiration, reduce arsenic-containing iron oxides, and release absorbed As(V). Furthermore, they can also reduce As(V) to As(III) and thus restore arsenic release (Islam et al., 2004; Ohtsuka et al., 2013). Although the abundance of *Geobacter* in the Nitrastur groundwater was relatively low, the possible contribution of this genus to arsenic conversion cycles would need to be analyzed in greater depth.

Iron oxides and hydroxides, as well as organic matter from coal residues from nearby coal washing plants that were used as filler material, are widespread at the Nitrastur site. Polycyclic aromatic hydrocarbons (PAHs) originating from incomplete combustion of organic fuel during pyrite ore smelting at the nearby metallurgic plant and accumulating in pyrite ash, are found extensively in the soils of Nitrastur (Gallego et al., 2016). The seepage of these compounds into groundwater provides abundant potential substrates for hydrogen generation under both microaerophilic and anaerobic conditions, such as those that occur in groundwater at Nitrastur. The existence of basal or higher levels of hydrogen before and after treatment with nZVI, together with the presence of sulfur in distinct oxidation states, and electron donors/acceptors, such as nitrate, would thus promote the development of microaerophilic bacteria and/or facultative and strict anaerobic bacteria before and after treatment with nZVI. *Hydrogenophaga* and *Desulfovibrio* are indicative of these changes. The low level of nitrate present in the groundwater at the end of the experiment (50 μgL^–1^) with respect to that before the addition of nZVI (an average of 1.092 mgL^–1^) (Table 1) may point to a high turnover of the nitrogen forms, possibly driven by the presence of additional electron donor compounds, such as those related to reduced forms of sulfur. These conditions would enhance the potential denitrifying activity of *Dechloromonas* and/or *Sulfuricurvum* bacteria, which showed a marked increase in population in the anaerobic environment promoted by nZVI, while the abundance of other hydrogen-oxidizing bacteria such as *Hydrogenophaga* significantly decreased. Under reducing conditions, sulfate-reducing bacteria (SRB) can modulate the concentration of metallic pollutants and arsenic through the use of organic metabolites (or nZVI, as a direct source of Fe(II) and hydrogen reductants) and the generation of biogenic pyrite, which can remove dissolved arsenic from contaminated groundwater through adsorption and co-precipitation (Saunders et al., 2018).

Another interesting aspect to consider in the groundwater of Nitrastur is the amount of dissolved organic carbon (Table 1), which indicates an oligotrophic environment and therefore determines the characteristics of the bacteria present. Such characteristics include the formation of biofilms, which can develop on sediments and in the form of aggregates (microbial “mobile biofilms”) adapted to the hydrodynamic regime of groundwater and the available surface (Besemer, 2015) (Supplementary Material SM2). These biofilms may contain insoluble adhered organic matter, such as the PAHs mentioned above and/or metals (Ancion et al., 2013), on which facultative oligotrophic bacteria, including *Hydrogenophaga*, *Clostridium* and other producers of exopolysaccharides, develop oligotrophic biofilms (Niederdorfer et al., 2017; Jewell et al., 2017). Given this observation and the fact that biofilms established under oligotrophic conditions preferentially host populations with chemoautotrophic energy metabolism based on hydrogen oxidation (Wu X. et al., 2017), further studies of the groundwater from Nitrastur are needed to explore the significance of these structures in bacteria-nZVI interactions and the biogeochemical cycles determined by the nanoparticles. The information obtained will allow a deeper understanding of the extraordinary adaptations and metabolic networks of bacteria in these complex contaminated brownfield sites, and such knowledge would contribute to optimizing remediation treatments on a rational basis. Despite this and based on the data already available, a possible improvement in the remediation strategy would be the use of organic amendments that stimulate the growth of facultative/anaerobic SRB bacteria that participate in the sequestration of arsenic (Saunders et al., 2018; Zacarías-Estrada et al., 2020). However, in this case, it would be necessary to maintain an available proportion of Fe(II) in the groundwater for iron sulfide formation, since an excess of dissolved H_2_S may produce more soluble thioarsenic compounds (Saunders et al., 2018 and references therein).

## Conclusion

It is difficult to predict the performance of remediation treatments in the field because of the many biogeochemical parameters can influence the final result. In this regard, the study of the cultured and total bacterial populations of the groundwater at the Nitrastur site has allowed us to propose reliable hypotheses regarding the response of the microbial communities to the treatment with nZVI and the contaminants present, in this case to arsenic. The microbial communities in the groundwater examined showed adaptation to the microaerophilic and anaerobic environmental conditions, present before and after the addition of nZVI. We propose that changes in taxonomic composition and population diversity that accompanied the addition of nZVI reflect a resilience mechanism of the bacterial communities rather than a direct and specific toxic effect. This adaptation allows the development of a new functional biogeochemical ecosystem in response to the new environmental conditions produced by the treatment, including changes in pH and oxidation-reduction potential as well as the predominance of anaerobic conditions. In the context of remediation treatments, our results also point to the importance of understanding the metabolic interactions of the microorganisms with iron, sulfur, arsenic, and probably nitrate, and their related adsorption processes in the oligotrophic groundwater of interest. The detailed study of these interactions in this and other remediation studies will pave the way for specific strategies to achieve more effective and long-lasting large-scale treatments of sites with a heavy arsenic load.

## Data Availability Statement

The raw data supporting the conclusions of this article will be made available by the authors, without undue reservation.

## Author Contributions

AC carried out the experimental work and data analysis and drafted the manuscript. AP carried out the molecular and statistical analyses and reviewed the data. DB, NO, and ER-V performed field-work and the chemical characterization of groundwater samples and checked the chemical data. JG and HS contributed to the experimental design, data analysis, and revision of the manuscript. AIP contributed to the conception, design, and supervision of the study, and the writing of the final version. All the authors approved the submitted version.

## Conflict of Interest

The authors declare that the research was conducted in the absence of any commercial or financial relationships that could be construed as a potential conflict of interest.

## Footnotes

1 http://www.nanorem.eu/index.aspx

2 https://pubmed.ncbi.nlm.nih.gov/2231712/

3 https://www.arb-silva.de/
