# Supplementary material for: Effects of in situ Remediation With Nanoscale Zero Valence Iron on the Physicochemical Conditions and Bacterial Communities of Groundwater Contaminated With Arsenic
Source: Front Microbiol. 2021 Mar 17;12:643589. doi: 10.3389/fmicb.2021.643589 (PMC8010140; doi:10.3389/fmicb.2021.643589)
Supplement: Supplementary file 1 [file Data_Sheet_1.PDF]

Supplementary Material

Effects of in situ remediation with nanoscale zero valence iron on the physicochemical conditions and bacterial communities of groundwater contaminated with arsenic.

Ana Castaño, Alexander Prosenkov, Diego Bragaño, Nerea Otaegui, Eduardo Rodríguez-Valdés, Herminio Sastre, José Luis Rodríguez Gallego, Ana Isabel Peláez*

*** Correspondence:** Corresponding Author: pelaezana@uniovi.es

SM 1: Oligonucleotide primers used for the detection of arsenic resistance genes

Target	Primer name	Primer sequence (5´-3')	Amplicon size (bp)	Reference
acr3.1	Aacr1F	GCGATGGCCAGCTCRAARTTRTT	≈750	Fahy et al., 2015
	Aacr2R	GGCCTGATCGTNATGATGTAYCC		
acr3.2	dacr5F	CGGCCACGGCCAGYTCRAARAARTT	≈750	Fahy et al., 2015
	dacr4R	TGATCTGGGTCATGATCTTCCCVATGMTGVT		
arsB	AarsB1F	GTACACCACCAGRTACATNCC	≈750	Fahy et al., 2015
	AarsB1R	GAACATCGTCTGGAAYGCNAC		
aioA1*	aroA-1F	GTSGGBTGYGGMTAYCABGYCTA	≈500	Inskeep et al., 2007
	aroA-1R	TTGTASGCBGGNCGRTTRTGRAT		
aioA2*	aroA-2F	GTCGGYTGYGGMTAYCAYGYYTA	≈500	Inskeep et al., 2007
	aroA-2R	YTCDGARTTGTAGGCYGGBCG		
arrA	ArrA-CVF1	CACAGCGCATCTGCGCCGA	≈330	Mirza et al., 2017
	ArrA-CVR1	CCGACGAACTCCYTGYTCCA		

*Proposed nomenclature for the large subunit of the arsenite oxidase (Lett et al., 2012).

PCR conditions were those described previously (Fahy et al., 2015; Mirza et al., 2017), with some modifications: for *aioA1*, denaturation was at 95ºC for 45sec, annealing at 50ºC-45ºC (-0,5ºC for cycle), extension at 72ºC for 50sec for 9 cycles and denaturation at 95ºC for 45 sec, annealing at 46ºC for 45 sec, extension at 72ºC for 50 sec for 24 cycles; for *aioA2*, denaturation at 92ºC for 1 min, annealing at 50ºC for 1 min 30 sec and extension at 72ºC for 1 m for 35 cycles; for *arrA*, denaturation at 95ºC for 30 sec, annealing at 50ºC for 30 sec and extension 72ºC for 30 sec for 35 cycles.

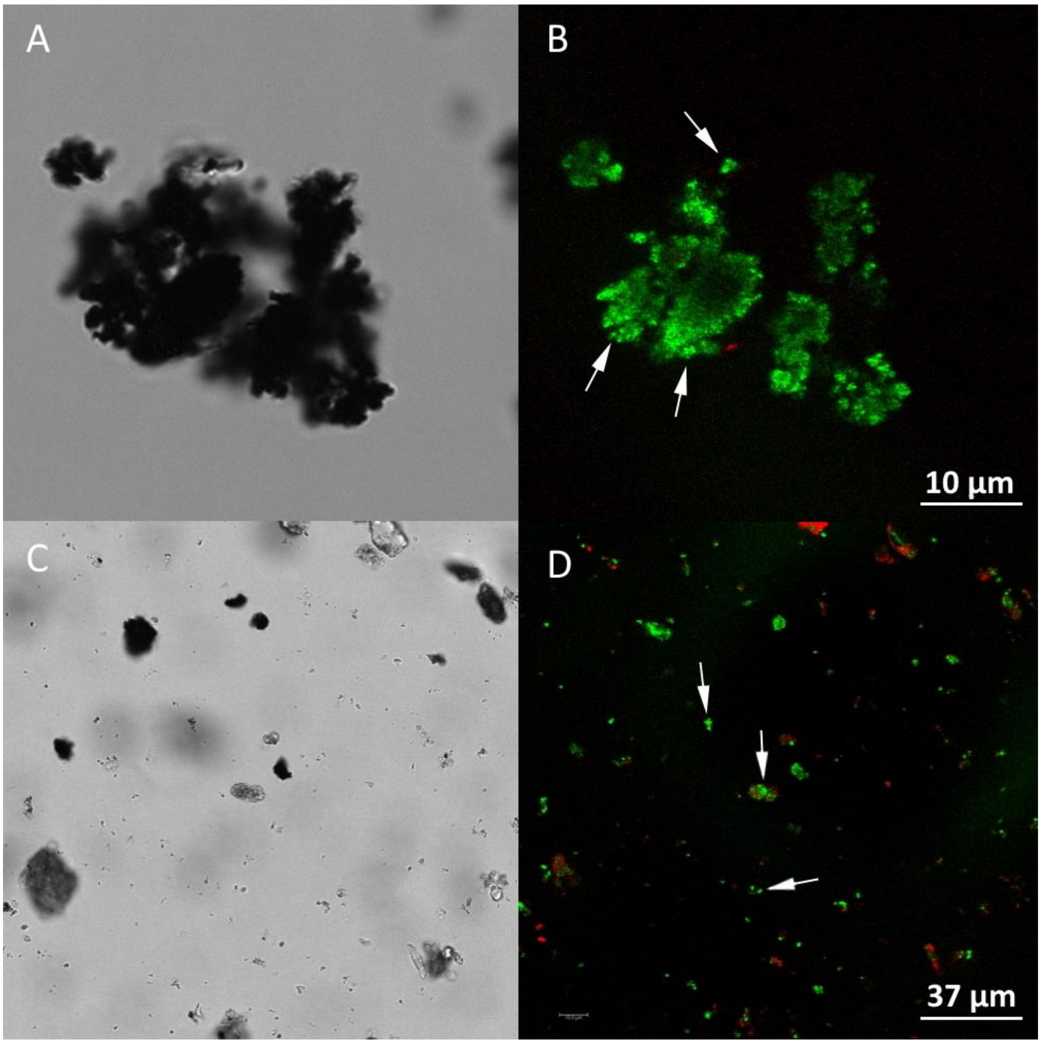

SM 2: Bacterial viability of the Nitrastur groundwater samples from CP-5 (A, B) and CP-6 wells (C, D), observed in the confocal laser scanning microscope. Samples were obtained 24 hours after the addition of nZVI and stained with PI and SYTO 9 (B, D). Bacteria with intact cell membranes appear fluorescent green, whereas membrane-compromised bacteria would appear red. The same images, obtained in differential interference contrast mode (A, C), allow to observe the structure of the sample. In image B, the bacteria appear attached to nanoparticle aggregates, while in image D appear free or attached to small particles in the aqueous suspension (arrows)

SM 3: Identification of the bacteria isolated from the groundwater sampling wells of Nitrastur, at the times indicated (shaded boxes)

Isolate Code	Most closely related species (NCBI accession number)	% ID	Taxonomic level (Phylum/Class)	Sample well and time (days)																			
				CP-2					CP-4					CP-5					CP-6				
				0	3	19	90	184	0	3	19	90	184	0	3	19	90	184	0	3	19	90	184
**A1**	*Pedobacter composti* strain TR6-06 (NR_041506.1)	98	*Bacteroidetes/ Sphingobacteria*	■													■			■		■	
**A2**	*Dietzia natronolimnaea* strain DSM 44860 (NR_116683.1)	97	*Actinobacteria/ Actinobacteria*																				■
**C5**	*Nocardia soli* strain SF 3 (MG051299.1)	98	*Actinobacteria/ Actinobacteria*								■												
**D4**	*Kocuria rhizophila* strain XFB-AX (KC429601.1)	99	*Actinobacteria/ Actinobacteria*										■	■									
**D5**	*Rhodanobacter spathiphylli* strain B39 (NR_042434.1)	97	*Proteobcteria/ Gammaproteobacteria*															■					■
**D6**	*Bacillus subtilis* strain SR2 (MT002768.1)	99	*Firmicutes/ Bacilli*		■				■												■		■
**D7**	*Microbacterium sp. strain PRA7 (MH412997.1)*	98	*Actinobacteria/ Actinobacteria*	■																			
**D8**	*Microbacterium oxydans* strain AU H10 (KU925166.1)	99	*Actinobacteria/ Actinobacteria*			■									■						■		
**D9**	*Hydrogenophaga* sp. BA-156 (HF548379.1)	98	*Proteobcteria/ Betaproteobacteria*	■			■		■				■			■	■		■			■	■
**D10**	*Microcella putealis* strain CV-2 (NR_042276.1)	98	*Actinobacteria/ Actinobacteria*					■				■						■					■
**D12**	*Sphingopyxis* sp. strain X20 (KM365437.1)	99	*Proteobacteria/ Alphaproteobacteria*				■																■
**D18**	*Agrococcus jenensis* strain RBE2CD-127 (EF111260.1)	98	*Actinobacteria/ Actinobacteria*								■	■											
**D20**	*Microbacterium* sp. 9_41 (HF954520.1)	97	*Actinobacteria/ Actinobacteria*			■																	
**D22**	*Sphingobium limnetium* strain 301 (NR_109484.1)	99	*Proteobacteria/ Alphaproteobacteria*																			■	
**E5**	*Planomicrobium okeanokoites* strain NBRC 12536 (NR_113593.1)	99	*Firmicutes/ Bacilli*	■	■			■									■			■			
**E6**	*Arthrobacter chlorophenolicus* strain A6 (NR_074518.1)	94	*Actinobacteria/ Actinobacteria*																■				
**E10**	*Dyadobacter soli* strain MJ20 (NR_117263.1)	99	*Bacteroidetes/ Cytophagia*		■																		
**E11**	*Dermacoccus nishinomiyaensis* strain DSM 20448 (NR_044872.1)	99	*Actinobacteria/ Actinobacteria*													■							

**F2**	*Massilia* sp. strain SKI 14.14 (MW037091.1)	98	*Proteobacteria/ Betaproteobacteria*																				
**F3**	*Pseudomonas parafulva* strain NBRC 16636 (NR_040859.1)	99	*Proteobacteria/ Gammaproteobacteria*																				
**F4**	*Bacillus cereus* ATCC 14579 (NR_074540.1)	98	*Firmicutes/ Bacilli*																				
**F5**	*Psychrobacter faecalis* T30 (MT313131.1)	99	*Proteobacteria/ Gammaproteobacteria*																				
**F6**	*Acidovorax* sp. TM17 (KC294123.1)	99	*Proteobacteria/ Betaproteobacteria*																				
**F7**	*Acinetobacter lwoffii* strain CIP 103579 (NR_104948.1)	99	*Proteobacteria/ Gammaproteobacteria*																				
**F11**	*Bacillus simplex* strain DSM 1321 (NR_042136.1)	99	*Firmicutes/ Bacilli*																				
**F17**	*Pseudomonas peli* strain R-20805 (NR_042451.1)	98	*Proteobacteria/ Gammaproteobacteria*																				
**F19**	*Arthrobacter globiformis* strain JCM 1332 (NR_112192.1)	99	*Actinobacteria/ Actinobacteria*																				
**F20**	*Pseudomonas brenneri* strain 4G513 (KY939742.1)	98	*Proteobacteria/ Gammaproteobacteria*																				
**F22**	*Aminobacter* sp. strain FW305-C-52 (MK402949.2)	98	*Proteobacteria/ Alphaproteobacteria*																				
**F30**	*Ideonella paludis* strain KBP-31 (NR_148652.1)	96	*Proteobacteria/ Betaproteobacteria*																				

SM 4: Arsenic tolerance of the bacteria isolated from the groundwater sampling wells of Nitrastur and presence of arsenic-resistance genes (shaded boxes).

Isolate Code (see SM2)	As(III) Concentration (mM)	As(V) Concentration (mM)	Resistant genes				
			acr3.1	*acr3.2*	*arsB*	*aio*	*arrA*
A1	2	50	■			■	
A2	2	100					
C5	2	100					
D4	2	100	■		■		■
D5	2	1				■	
D6	2	20					
D7	5	100					■
D8	20	100					■
D9	2	20		■		■	
D10	5	100					
D12	15	100	■			■	■
D18	2	100	■		■		
D20	2	20					
D22	2	100	■	■		■	
E5	5	100			■		
E6	2	100	■	■			
E10	2	50	■			■	■
E11	2	100	■	■			
F2	0	100				■	
F3	10	100			■	■	
F4	5	100			■		■
F5	2	100		■			
F6	2	100		■	■		■
F7	0	100					
F11	2	100					
F17	0	0		■			■
F19	2	100	■			■	■
F20	10	100			■	■	
F22	2	100		■		■	
F30	0	0		■	■	■	

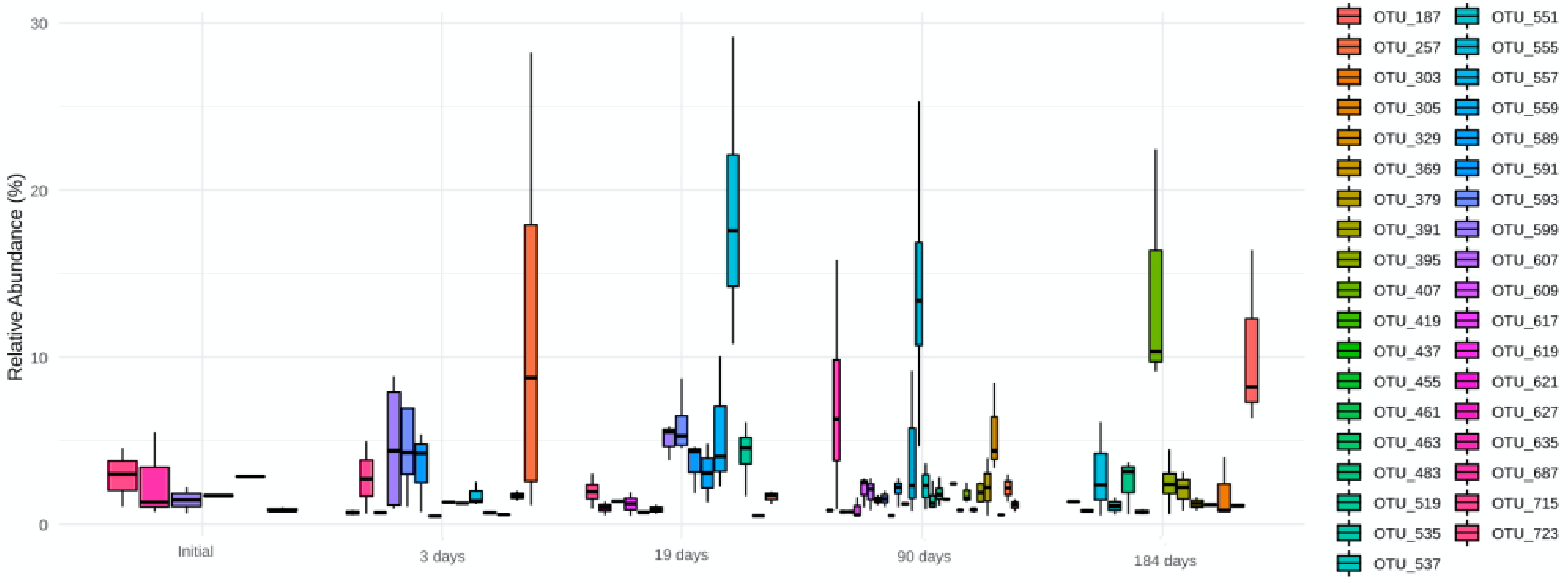

SM 5: Most abundant operational taxonomic units (OTUs) from ARISA analysis, contributing to differences between the bacterial communities according to sampling time.

SM 6: Percentage of 16S rDNA sequences assigned to the different phyla in the four Nitrastur groundwater wells (CP-2, CP-4, CP-5 and CP-6), before adding nZVI (0), and at the end of the treatment (184 days).

Phylum	CP-2.0	CP-2.184	CP-4.0	CP-4.184	CP-5.0	CP-5.184	CP-6.0	CP-6.184
Proteobacteria	71,2	66,2	94,5	64,6	85,2	71,9	90,6	78,9
Firmicutes	0,4	31,3	0,2	18,1	0,2	19,3	0	11,6
Bacteroidetes	23,7	1,8	5	7,1	13,7	5,3	5,7	3,5
Spirochaetae	0,02	0,27	0	3,7	0,01	1,7	0	2,9
Ignavibacteriae	1,5	0,05	0,02	0,68	0,1	0,48	0,72	0,79
Actinobacteria	0,19	0,08	0,1	2,13	0,11	0,32	0,04	0,54
Elusimicrobia	0,08	0,04	0	1,67	0	0,28	0,03	0,47
Chloroflexi	0,41	0,03	0,01	0,93	0,08	0,33	0,77	0,56
Acidobacteria	0,89	0,06	0,1	0,27	0,17	0,11	0,87	0,2
Omnitrophica	0,6	0,12	0	0,46	0,17	0,21	0,15	0,37
Planctomycetes	0,32	0	0,03	0,14	0,1	0,02	0,72	0,06
Nitrospirae	0,55	0,02	0,02	0,12	0,11	0,05	0,37	0,08

SM 7: Percentage of 16S rDNA sequences assigned to the different genera in the four Nitrastur groundwater wells (CP-2, CP-4, CP-5 and CP-6), before adding nZVI (0), and at the end of the treatment (184 days).

Genus	Phylum/class	CP-2.0	CP-2.184	CP-4.0	CP-4.184	CP-5.0	CP-5.184	CP-6.0	CP-6.184
Desulfovibrio	delta Proteobacteria	0	55,7	0	54,8	0	70	0	56,6
Acinetobacter	gamma Proteobacteria	9	0	83,4	0,6	41,9	0	29,2	0
Desulfoporosinus	Firmicutes	0	30,7	0	14	0	20,1	0	9,9
Perlucidibaca	gamma Proteobacteria	13,1	0	0	0	1,8	0	40,7	0
Hydrogenophaga	beta Proteobacteria	8,9	5,8	4,7	7,1	8,2	0	18,7	1,1
Uncultured *Sphingobacteria*	Bacteroidetes	40,6	0	0	0,1	10,5	0	0	0
Dechloromonas	beta Proteobacteria	6,1	5,9	0,4	9,8	3,4	0,4	1,1	12,8
Pseudomonas	gamma Proteobacteria	0,8	0	1,5	0	25,7	0	0,9	0
Sulfuricurvum	epsilon Proteobacteria	3,9	0	0	0,1	0	3,9	0,5	13,7
Novosphingobium	alpha Proteobacteria	3,8	0	5,0	0	4,2	0	3,6	0
Flavobacterium	Bacteroidetes	5,4	0	3,1	0,1	2,4	0	2,8	0
Uncultured *Bacteroidetes*	Bacteroidetes	3,3	1,5	0	3,1	0,5	2,9	0,3	1,5
Uncultured *Spirochaetes*	Spirochaetes	0	0	0	5,2	0	2	0	3,3
Leadbetterella	Bacteroidetes	4,8	0	1,8	0	1,3	0	2	0
Geobacter	delta Proteobacteria	0	0	0	4,9	0	0,5	0	1

REFERENCES

Fahy A, Giloteaux L, Bertin P, Le Paslier D, Médigue C, Weissenbach J, Duran R, Lauga B. (2015). 16S rRNA and As-Related Functional Diversity: Contrasting Fingerprints in Arsenic-Rich Sediments from an Acid Mine Drainage. Microb Ecol. 70:154-67. doi: 10.1007/s00248-014-0558-3.

Inskeep WP, Macur RE, Hamamura N, Warelow TP, Ward SA, Santini JM. (2007). Detection, diversity and expression of aerobic bacterial arsenite oxidase genes. Environ Microbiol. 9:934-43. doi: 10.1111/j.1462-2920.2006.01215.x.

Lett MC, Muller D, Lievremont D, Silver S, Santini J (2012). Unified nomenclature for genes involved in prokaryotic aerobic arsenite oxidation. J Bacteriol 194: 207–208.

Mirza, B. S., Sorensen, D. L., Dupont, R. R., & McLean, J. E. (2017). New Arsenate Reductase Gene (arrA) PCR Primers for Diversity Assessment and Quantification in Environmental Samples. Applied and Environmental Microbiology, 83(4), e02725-16. https://doi.org/10.1128/AEM.02725-16
